# Correlation of the anterior ocular segment biometry with HbA1c level in type 2 diabetes mellitus patients

**DOI:** 10.1371/journal.pone.0191134

**Published:** 2018-01-11

**Authors:** Abd-Rashid Suraida, Mohtar Ibrahim, Embong Zunaina

**Affiliations:** 1 Department of Ophthalmology, School of Medical Sciences, Universiti Sains Malaysia, Kubang Kerian, Kelantan, Malaysia; 2 Hospital Universiti Sains Malaysia, Jalan Raja Perempuan Zainab II, Kubang Kerian, Kelantan, Malaysia; University of Florida, UNITED STATES

## Abstract

**Objectives:**

To compare the anterior ocular segment biometry among Type 2 diabetes mellitus (DM) with no diabetic retinopathy (DR) and non-proliferative diabetic retinopathy (NPDR), and to evaluate the correlation of anterior ocular segment biometry with HbA1c level.

**Methods:**

A cross-sectional study was conducted in Hospital Universiti Sains Malaysia, Kelantan from November 2013 till May 2016 among Type 2 DM patients (DM with no DR and DM with NPDR). The patients were evaluated for anterior ocular segment biometry [central corneal thickness (CCT), anterior chamber width (ACW), angle opening distance (AOD) and anterior chamber angle (ACA)] by using Anterior Segment Optical Coherence Tomography (AS-OCT). Three ml venous blood was taken for the measurement of HbA1c.

**Results:**

A total of 150 patients were included in this study (DM with no DR: 50 patients, DM with NPDR: 50 patients, non DM: 50 patients as a control group). The mean CCT and ACW showed significant difference among the three groups (p < 0.001 and p = 0.015 respectively). Based on post hoc result, there were significant mean difference of CCT between non DM and DM with NPDR (mean difference 36.14 μm, p < 0.001) and also between non DM and DM with no DR (mean difference 31.48 μm, p = 0.003). The ACW was significantly narrower in DM with NPDR (11.39 mm SD 0.62) compared to DM with no DR (11.76 mm SD 0.53) (p = 0.012). There were no significant correlation between HbA1c and all the anterior ocular segment biometry.

**Conclusion:**

Diabetic patients have significantly thicker CCT regardless of retinopathy status whereas ACW was significantly narrower in DM with NPDR group compared to DM with no DR. There was no significant correlations between HbA1c and all anterior ocular segment biometry in diabetic patients regardless of DR status.

## Introduction

Diabetes mellitus (DM) is a group of metabolic diseases characterized by hyperglycemia [fasting plasma glucose ≥126 mg/dl (7.0 mmol/l) or 2-h plasma glucose ≥200 mg/dl (11.1 mmol/l)] resulting from defects in insulin secretion, insulin action, or both [1]. Type 1 diabetes mellitus results from the body’s failure to produce insulin, and requires the person to inject insulin or wear an insulin pump. Type 2 diabetes mellitus results from insulin resistance, a condition in which cells fail to use insulin properly, sometimes combined with an absolute insulin deficiency. Chronic hyperglycemia leads to multiple organs damage especially the eyes, kidneys, nerves, heart, and blood vessels. Diabetic retinopathy (DR), nephropathy and peripheral neuropathy are the common complications of DM [1].

DR is one of the causes of blindness worldwide. There are many risk factors for DR. The duration of DM significantly associated with the development and severity of DR. Significant systemic risk factors include hypertension and high glycosylated haemoglobin A1c (HbA1c), systolic blood pressure, pulse pressure, serum lipoprotein level and body mass index. DR can be prevented by understanding the ocular conditions and early detection. Therefore, periodic eye examinations together with good glycemic control are the initial steps to reduce the risk of ocular complications. Other measure include stabilisation of systemic risk factors such as hypertension, hyperlipidaemia, and anaemia [2, 3].

DR is predominantly a microvascular disease. It affects the smaller vessels by causing multilayering of the basement membrane and degeneration of the endothelial cells and the pericytes that lead to capillary occlusion and leakage. DR is divided into two main groups namely the nonproliferative diabetic retinopathy (NPDR) and the proliferative diabetic retinopathy (PDR).

Other than retina, diabetic also may affect anterior segment structures of the eye such as cornea, iris, ciliary processes, lens, anterior chamber and posterior chamber. The anterior chamber composed of posterior surface of the cornea, the anterior surface of the iris and the sclerocorneal angle, where the trabecular meshwork, the scleral spur, the ciliary body, and the iris root located. The narrowest portion of the anterior chamber is at the angle. Scleral spur is visible as an inward projection of the sclera at the junction between the inner scleral and corneal curvatures. Anterior chamber width (ACW) is the distance between left scleral spur and right scleral spur [4]. Changes of the anterior segement structures include vacuolation of the iris pigment epithelium, thickening of the basement membrane of the ciliary processes, thickening of the cornea and cataract formation. Early detection of any changes in anterior ocular segment biometry will help for early intervention and provide effective treatment in order to reduce the risk of vision loss.

Several studies have reported thickening of central corneal thickness (CCT) in people with diabetes [5,6,7,8]. The basis for the association is unknown but one of the postulation is hyperglycaemia may cause corneal endothelial dysfunction with resultant stromal hydration and swelling of the cornea with some suggesting that this may be one of the earliest changes detectable in the diabetic eye [9,10]. However, other studies [11,12] have failed to demonstrate significant differences in corneal thickness between Type 2 DM subjects as compared to age-matched control subjects.

According to Nongpiur *et al* [13] through their cross section study done in Singapore, they found that ACW was smaller in women, Chinese persons, and older persons, and was associated with narrow angles thus suggested it as a risk indicator for angle closure. A smaller ACW also implies a smaller anterior chamber volume, and this may facilitate the angle-crowding mechanism.

However one study done by Xu *et al* [14] which was a population-based study on Chinese adults living in the greater Beijing area suggests that a shallow anterior chamber and a narrow anterior chamber angle (ACA) are associated with short body stature, higher age, female gender, higher CCT, nuclear cataract, hyperopia, small optic disc, and chronic angle closure glaucoma (ACG) and not associated with DR.

HbA1c is regarded as the gold standard indicator for glycemic control in diabetic patients. It is formed in a non-enzymatic glycation pathway by haemoglobin’s exposure to plasma glucose. Normal levels of glucose produce a normal amount of HbA1c. As the average amount of plasma glucose increases, the fraction of HbA1c increases in a predictable way. This serves as a marker for average blood glucose levels over the previous months prior to the measurement. The level of HbA1c is proportional to both the average glucose concentration and the life span of the red blood cell in the circulation. The measurement of HbA1c has therefore been accepted for the clinical management of diabetes through routine monitoring. Guidelines now recommend that attaining HbA1c level of 6.5% or below could significantly reduce the risk of developing the various complications of diabetes [1]. HbA1c measurement reflects the glucose level over the period of 2–3 months. Therefore, complications and progression of diabetic is related to the status of blood sugar control [15].

Study suggested that variations in glucose levels of at least up to 3 months (HbA1c) probably affect CCT to a greater extent than short-term fluctuations of glucose levels other than severity of retinal complications [16]. While Lee et al [9] reported that increased corneal thickness correlated with duration of disease of greater than 10 years. However, a study done by El-Agamy and Al-Subaie S showed there was no correlation between CCT and HbA1c [8].

Beside that, a study done in a population of Singapore Malays found that there was no significant association of anterior angle parameter and HbA1c [17].

Nowadays, in the presence of new technology of imaging devices such as anterior segment optical coherence tomography (AS-OCT), wide variety of corneal and anterior segment conditions can be evaluated and measured quantitatively. This is useful infacilitating and enhancing diagnosis, clinical evaluations, patient management and therapeutic decisions [18].

Changes in anterior ocular segment biometry may be some of the earliest clinically detectable changes in the diabetic eye. Although differences between diabetic and non-diabetic eyes have been elucidated, the association between the severity of DR and the anterior segment changes has not been thoroughly investigated. A positive correlation between the severity of the DM and anterior ocular segment biometry may provide early clues of the potential risks of retinal complication in diabetic individuals.

The objective of this study is to compare the anterior ocular segment biometry involving CCT, ACW, angle opening distance (AOD) and ACA between Type 2 DM with no DR and NPDR. The second objective is to determine the correlation between anterior ocular segment biometry and HbA1c level.

## Methods

### Participants

A cross-sectional study was conducted at Hospital Universiti Sains Malaysia among Type 2 DM patients. Type 2 DM patients with NPDR, and DM with no DR that attending Diabetic Clinic and Eye Clinic from November 2013 till May 2016 were recruited in the study.

Sample size was calculated by using power and sample size (PS) software. Formula t test was used to determine the sample size for comparison of the mean of anterior ocular segment biometry among Type 2 DM patients between NPDR and no DR. Whereas, Cohen Table [19] was used for determination of sample size for the correlation between anterior ocular segment biometry and HbA1c among Type 2 DM patients. Based on the calculation, 50 patients for each group were required. Informed consent was obtained from the patient before conducting the study.

The inclusion criteria for the patients in the study group were: (i) All Type 2 DM patients with no DR or DM with NPDR, (ii) Duration of DM from the time of diagnosis at least 5 years, (iii) The age of the patients were above or equal to 40 years old to 65 years old. Eyes with proliferative diabetic retinopathy (PDR), primary or secondary glaucoma, corneal pathology, contact lens wearer and post intraocular surgery or ocular trauma were excluded from the study. Patients with medical problems related to water retention such as chronic renal failure, congestive cardiac failure, and nephrotic syndrome were also excluded.

Non-diabetic patients with normal ocular findings except minimal cataract, non-contact lens wearer, and age between 40–65 years old were selected as a control group. Fasting plasma glucose were done to screen for the undiagnosed DM. As in study group, non-diabetic patients with medical problem related to water retention such as chronic renal failure, congestive cardiac failure, and nephrotic syndrome were excluded.

### Study procedure

The detail of all the procedures were explained to all patients and control group. The demographic and clinical data were collected from the patient’s medical records. Then followed by examination of the fundus and measurements of the anterior ocular segment biometry.

#### Fundus examination

The pupils were dilated using dilating eye drops (mydriacyl 1%, phenylephrine 2.5%). Examination of the fundus was done by principle investigator using slit lamp biomicroscope with 90 dioptre (D) condensing lens or binocular indirect ophthalmoscope with 20 D condensing lens. Fundus photograph was taken by trained medical operator for grading of DR. The status of DR was classified based on Early Treatment Diabetic Retinopathy Study (ETDRS) [20] by one identified ophthalmologist. The patient was given next appointment (within one week) for anterior ocular segment biometry measurement.

#### Anterior ocular segment biometry measurement

**Fig 1:**
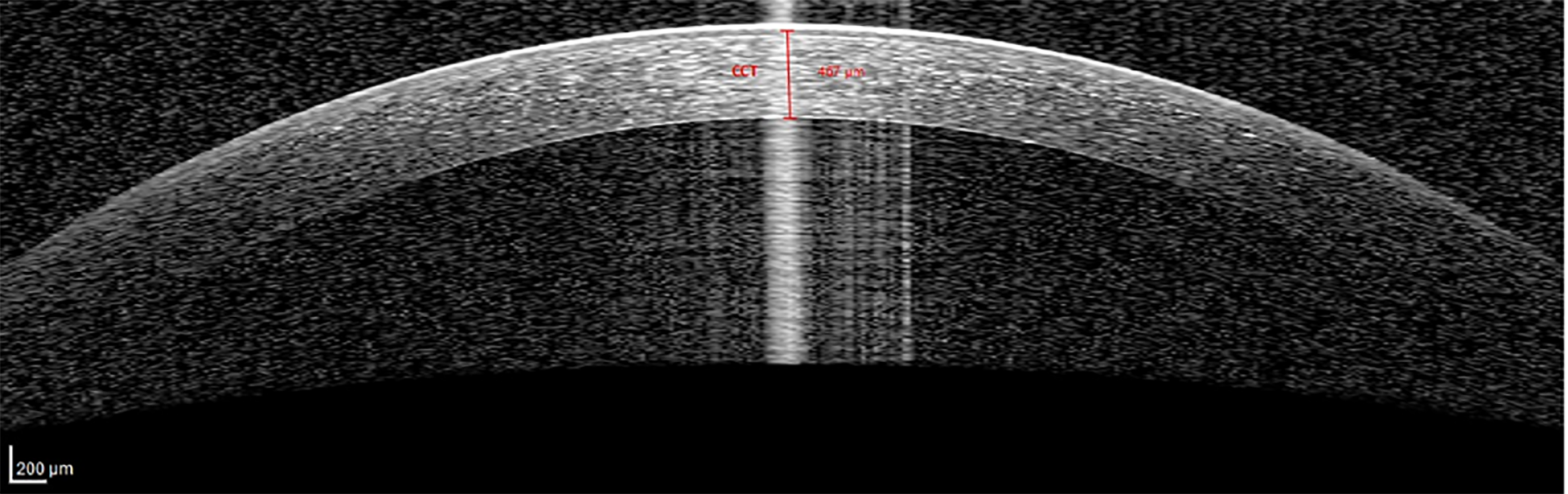
Schematic figure illustrating CCT from AS-OCT. Abbreviation: CCT: central corneal thickness, AS-OCT: Anterior Segment Optical Coherence Tomography.

**Fig 2:**
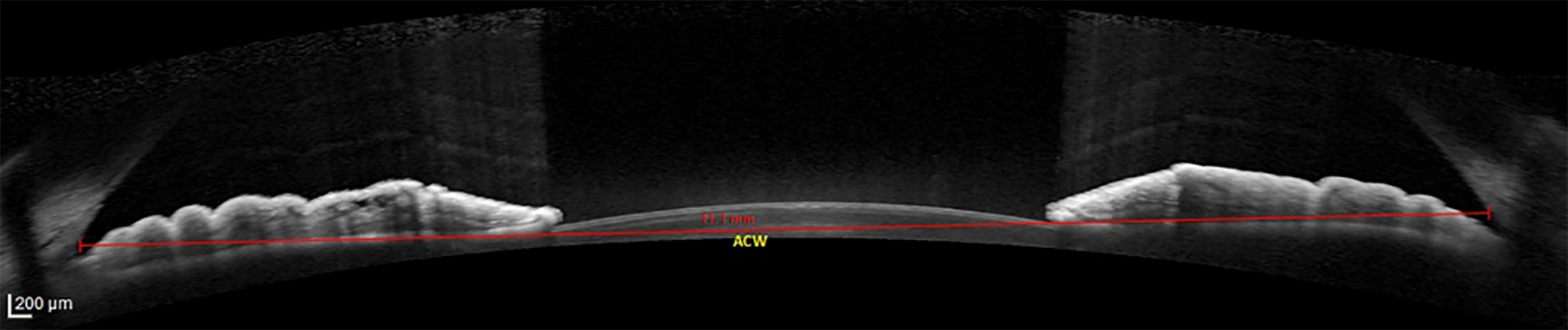
Schematic figure illustrating ACW from AS-OCT. Abbreviation: ACW: anterior chamber width, AS-OCT: Anterior Segment Optical Coherence Tomography.

**Fig 3:**
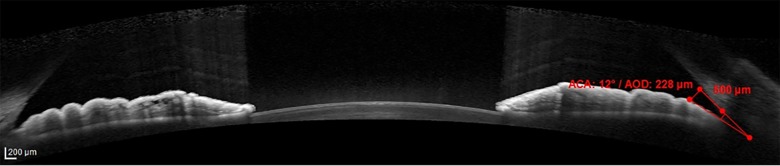
Quantitative measurements of ACA and AOD by AS-OCT. Abbreviation: ACA: anterior chamber angle, AOD: angle opening distance, AS-OCT: Anterior Segment Optical Coherence Tomography.

The anterior ocular segment biometry measurement was done using AS-OCT (Spectralis Heidelberg, Germany) by one identified trained medical operator which was blinded to the status of DM (study and control group) and also the staging of DR of the diabetic patients. In cases where both eyes of a single patient fulfilled the inclusion criteria, the right eye was selected for measurement of anterior ocular segment biometry. The subject was instructed to rest the forehead on the headrest and focus on the blue light that appears from the camera. Once a good image of anterior segment obtained, the image was captured by the operator. The anterior ocular segment biometry that was measured were CCT, ACW, AOD and ACA. The CCT is the thickness of the cornea at its centre (Fig 1). The CCT of a normal and healthy cornea ranges from 450 to 650 μm [21]. ACW is the distance between left scleral spur and right scleral spur (Fig 2). Scleral spur is visible as an inward projection of the scleral at the junction between the inner scleral and corneal curvatures. ACW in normal population range 11.48 mm to 13.54 mm [4]. AOD is defined as the length of the line, perpendicular to the corneal endothelial surface, from a point on the endothelial surface 500 μm (AOD 500) or 750 μm (AOD 750) anterior to the scleral spur to the iris surface. AOD 500 was measured in this study (Fig 3). ACA is defined in degrees, in which the angle recess forms the apex and the two sides of the angle are formed by drawing the lines through the points defining the AOD. AOD 500 was used in this study (Fig 3). The measurement of anterior ocular segment biometry was analysed by principle investigator.

#### HbA1c measurement

After completing the anterior ocular segment biometry measurement, 3 ml of venous blood was obtained and kept in a universal bottle with ethylenediaminetetraacetic acid (EDTA) for determination of serum HbA1c level. The percentage of the A1c component of HbA1c was assessed by high performance liquid chromatography as described in detail previously [22] with the range of normal value is between 4.5% to 6.5% [23].

### Ethical approval

This study received ethical approval from local ethics committee {Ref: USMKK/PPP/JEPeM [283.3.(16)]} of Universiti Sains Malaysia.

### Statistical analysis

Statistical Package for Social Sciences (SPSS) version 21.0 were used for analysis of data. Mean values and standard deviation (SD) were used for descriptive analyses. One-way Anova test was used to compare the mean age and mean of anterior ocular segment biometry between the three groups. Whereas Pearson Chi square test was used to compare gender and ethnicity. The mean of HbA1c was tested by Independent t-test. Pearson correlation was used to determine the correlation between anterior ocular segment biometry and HbA1c. Correlation coefficient, r is a measure of degree of linear relationship between two numerical variables; r = 0 means no correlation, r = +1 means a perfect positive relationship and r = -1 means perfect negative relationship. The r grading are; 0: no correlation, < 0.25: poor, 0.26–0.50: fair, 0.51–0.75: good, and 0.76–1.0: excellent [24]. P values < 0.05 were taken as significant data.

## Results

A total of 150 patients were recruited in the study (50 patients in DM with no DR, 50 patients in DM with NPDR, and 50 patients in non DM as a control group). The mean age of all patients was 57.81 SD 7.59 years. There was no significant difference in mean age between the three groups, non DM: 59.24 SD 6.64 years, DM with no DR: 58.24 SD 7.50 years and DM with NPDR: 57.38 SD 7.73 years (p = 0.446) (Table 1). The percentage of male gender was highest in DM with no DR (58%), whereas female gender was highest among DM with NPDR group (74%). There was significant difference between gender and each group (p = 0.005). Most of the patients were Malay (94.0%), 8 patients (5.3%) were Chinese and only one patient (0.7%) was Siamese. There was no significant difference between ethnic and each group (p = 0.423). The level of mean HbA1c was significantly higher in DM with NPDR compared to DM with no DR (9.65% SD 2.57 and 8.26% SD 1.77 respectively) (p = 0.002) (Table 1).

**Table 1:** Distribution of age, gender, ethnic group and HbA1c.

Characteristics	Non DM	DM with no DR	DM with NPDR	P value
	n = 50	n = 50	n = 50	
Mean age (year) (Mean, SD)				
	59.24 (6.64)	58.24 (7.50)	57.38 (7.73)	0.446^a^
Gender (n, %)				
Male	20 (40.0%)	29 (58.0%)	13 (26.0%)	
Female	30 (60.0%)	21 (42.0%)	37 (74.0%)	0.005^b^
HbA1c (%)	NA	8.26 (1.77)	9.65 (2.57)	0.002^c^

^a^One-way ANOVA.

^b^Pearson Chi sqquare test.

^c^Independent t-test.

P < 0.05 significant.

Abbreviation: DM: diabetes mellitus, DR: diabetic retinopathy, NPDR: non proliferative diabetic retinopathy, SD: standard deviation, NA: Not applicable, HbA1c: Glycosylated haemoglobulin

The distribution of mean for all anterior ocular segment biometry in three different groups is shown in Table 2. DM with no DR showed the highest means of all anterior ocular segment biometry except for the CCT between the three groups. There was significant difference of mean anterior ocular segment biometry for CCT (p < 0.001) and ACW (p = 0.015) between the three groups.

**Table 2:** Comparison of mean anterior ocular segment biometry between the groups.

Anterior segment ocular biometry	Mean (SD)			p value
	non DM (n = 50)	DM with no DR (n = 50)	DM with NPDR (n = 50)	
CCT (μm)	493.12 (67.08)	524.60 (28.74)	529.26 (33.88)	< 0.001
ACW (mm)	11.58 (0.74)	11.76 (0.53)	11.39 (0.62)	0.015
AOD-A (mm)	365.01 (112.81)	409.14 (187.91)	399.09 (175.73)	0.364
AOD-T (mm)	382.28 (157.62)	400.32 (211.68)	390.04 (163.81)	0.881
AOD-N (mm)	351.74 (95.35)	417.96 (181.01)	408.22 (199.99)	0.100
ACA-A (degree)	20.24 (5.90)	22.41 (7.81)	21.66 (8.16)	0.328
ACA-T (degree)	20.68 (8.22)	22.46 (9.16)	21.92 (7.98)	0.561
ACA-N (degree)	20.38 (7.49)	22.38 (7.49)	21.40 (9.60)	0.421

One-way Anova, p value <0.05 (significant).

Abbreviation: DM: diabetes mellitus, DR: diabetic retinopathy, NPDR: non proliferative diabetic retinopathy, SD: standard deviation, CCT: central corneal thickness, ACW: anterior chamber width, AOD: angle opening distance (A: average, T: temporal, N: nasal), ACA: anterior chamber angle (A: average, T: temporal, N: nasal).

Based on post hoc result (Table 3), there were significant mean difference of CCT between non DM and DM with NPDR (mean difference 36.14 μm, p < 0.001) and also between non DM and DM with no DR (mean difference 31.48 μm, p = 0.003). The ACW was significantly narrower in DM with NPDR (11.39 mm SD 0.62) compared to DM with no DR (11.76 mm SD 0.53) (p = 0.012).

**Table 3:** Post hoc comparison of mean CCT and ACW.

Anterior segment ocular biometry	Groups	Mean Difference (95% CI)	p value
CCT (μm)	non DM—DM with no DR	31.48	0.003
	non DM—DM with NPDR	36.14	< 0.001
ACW (mm)	DM with no DR—DM with NPDR	0.37	0.012

One-way Anova, p value <0.05 (significant).

Abbreviation: DM: diabetes mellitus, DR: diabetic retinopathy, NPDR: non proliferative diabetic retinopathy, CI: confidence interval, CCT: central corneal thickness, ACW: anterior chamber width.

There were no significant correlation between HbA1c and all the anterior ocular segment biometry among Type 2 DM.

## Discussion

Ocular complication secondary to DM is one of the causes of blindness despite good glycemic control and stabilization of systemic risk factors [2,3]. Although diabetic eye disease is often thought to refer merely to the retina, the anterior segment of the eye has also been shown to be affected in diabetes [5,9,25,26].

Optical coherence tomography (OCT) is being employed increasingly often to image pathology and surgical anatomy within the anterior segment, specifically in anterior chamber biometry, corneal pachymetric mapping, angle evaluation and high resolution cross-sectional imaging. The cross-sectional imaging capability of OCT is similar to ultrasound, but its higher resolution and non-contact measurement allows visualization of fine anatomic structures [27].

Previous studies have looked at the AS-OCT changes occurring in diabetic patients, and compared these with non DM patients. However, no previous study has attempted to compare the difference in AS-OCT findings among DM patients with NPDR and those with no DR. This present study was conducted to evaluate the anterior ocular segment biometry and describe the correlation of anterior ocular segment biometry with HbA1c among Type 2 DM with NPDR and with no DR.

A total of 150 patients were involved in this study. The mean age for our study was 57.81 (7.59) years. The age group in our study reflects higher prevalence of DR among older patients with Type 2 DM [28,29].

From our study, we found that most of our study populations were Malays, whereas the remainder was from non-Malay ethnics; Chinese and Siamese. According to The Malaysian National Health Morbidity Survey III (NHMS III) conducted in 2006 [30], the highest prevalence of diabetes nationwide was found to be among the Indians at 19.9% followed by 11.9% in the Malays and 11.4% in the Chinese. Malay is one of the racial in Malaysia and more dominant in Kelantan. Our finding reflects the normal racial distribution in Malaysia especially Kelantan.

Females comprised 58.7% of the sample. Although our study patients were age-matched, the proportion of females in the group of diabetic with NPDR differed from the other two groups. The Singapore Malay Eye Study showed a higher prevalence of DR in women as compared to men [31]. The reason for this is unclear and merits further investigation. However, other study has not shown a consistent pattern of gender variation in the prevalence of DR [32].

Several studies have compared anterior ocular segment biometry parameters among DM patients and non-DM patients. CCT has been shown to be thicker in DM patients than in non DM patients [5,10,33]. The basis for the association is unknown but one postulation is that hyperglycaemia may cause corneal endothelial dysfunction with resultant stromal hydration and swelling of the cornea, with some suggesting that this may be one of the earliest changes detectable in the diabetic eye [9,10,33]. Saini and Mittal [34] found that endothelial function was most adversely affected in patients with DR because Type 2 DM had significantly more compromised corneal endothelial function than controls.

According to Meyer et al [35] and Yee et al [36], who did experimental studies using mice and dogs, diabetes reduces the activity of sodium-potassium adenosine triphosphatase (Na^+^–K^+^ ATPase) of the corneal endothelium, thus causing permeability changes in the cornea and morphological changes such as decrease in the corneal endothelium density, a decrease in hexagonality, and an increase in the coefficient of variation for cell size.

With regards to CCT, our findings concur with previous studies, in which CCT was noted to be significantly thicker in DM patients than non DM patients, regardless of retinopathy status [5,9,10,33]. This suggests that the pathophysiological changes underlying diabetes itself are associated with changes in CCT, even in the absence of retinopathy.

DM may be a risk factor for ACG [25,26,37]. Shallower anterior chambers [25] and thicker lenses [26] in diabetic patients compared to non DM individual might explain the association of DM with ACG. A smaller anterior chamber volume also implies a smaller ACW, and this may facilitate the angle-crowding mechanism [13]. We postulated that a significant increase in oxidative stress as occurs in DR [38], also might contribute to the pathogenesis of primary ACG (PACG) [39,40].

There was a significant difference in the mean ACW between DM with NPDR and DM with no DR (p = 0.012), in which ACW was narrower in DM with NPDR compared to DM with no DR. Based on the ACW finding, diabetes itself, in the absence of DR, may not be a risk factor for PACG. Rather, the difference in ACW finding seen among DR patients may be due to the enhanced oxidative stress and/or reduction of antioxidant status [38,39,40], which may lead to thickening of the antero-posterior lens diameter, reduction in anterior chamber volume and narrowing of the angle. According to Agte and Tarwadi [41], the lens is the ocular structure most susceptible to oxidative damage. Unfortunately, due to lack of resources, we were unable to quantify this by measuring markers of oxidative stress. The effect on the cornea may not be as marked as the cornea is more resistant to oxidative stress compared to the lens.

This new finding of narrower ACW in DM with DR than in those without DR suggests that this parameter may be an adjunctive marker of current uncontrolled diabetic status. We postulate that in the presence of hyperglycaemia, oxidative stress results in an increase in lens thickness and thus a thicker lens vault. Lens vault was significantly associated with gonioscopy-defined occludable angles and might be important predictor for detecting angle closure [42].

Screening of patients with diabetes should include measurement of anterior segment parameters as this is a non-invasive, reliable and objective method of assessing the current diabetic control and the risk of complications. Future studies should include not only the parameters assessed in our study but also lens vault, as this has been shown to be a risk factor for angle closure.

We noted that the proportion of females in the group of DM with NPDR differed significantly from the other two groups, and that the former group was associated with a narrower ACW. The Singapore Malay Eye Study showed a higher prevalence of DR in women compared to men [31]. We postulate that the greater prevalence of DR in women may place them at risk of higher oxidative stress, which may have caused thickening of the antero-posterior diameters of the lens that lead to angle crowding. On the other hand, a population-based study on Chinese adults living in the greater Beijing area by Xu et al [14] found that a shallow and smaller anterior chamber are associated with short body stature, higher age, and female gender. In that same study by Xu et al [14], DR status was not associated with either a shallow anterior chamber or a narrow ACA. We are unable to conclude whether being female or having DM with NPDR have caused the narrower of ACW. More studies are required to elucidate the exact pathogenesis of these differences.

Previous studies found that AOD and ACA were narrower in DM compared to non-DM [13,25,26,]. Our findings contradict their studies, as we found no significant difference in AOD and ACA between DM and non DM patients. We believe that these differences may be due to fluctuations in anterior chamber angle parameters such as physiological dilatation and constriction of pupil.

Our results showed that ACW, AOD and ACA findings of the non DM group were closer to that of the NPDR group rather than those with DM but no DR. We are unsure regarding the possible mechanism underlying these findings but the similarities in anterior segment biometry parameters between the non DM group and the DM with NPDR groups may be due to the fact that the patients in the latter group may have had DR of a mild degree as well as a short duration of diabetes, in which the effect of oxidative stress is not yet observed.

Good glycaemic control is essential in preventing diabetic complications. The level of HbA1c provides information about the glycaemic control in DM patients during the preceding 2–3 months [43]. It is thus a clinical measure of chronic glycaemia [44]. Those with HbA1c values in the normal range have been shown to have a lower risk of diabetic complications [45]. Guidelines now recommend that attaining an HbA1c level of 6.5% or below could significantly reduce the risk of developing the various complications of diabetes [46].

The mean of HbA1c in DM with NPDR was 9.65% as compared to HbA1c level in DM with no DR, which was 8.26%. Either with or without DR, majority of the patients had HbA1c more than 6.5%. From our findings, mean HbA1c were consistent with other studies [30,47,48]. This shows that most patients in our study had poor control of their diabetes. This may be related to factors not assessed in this study, such as their level of education, awareness regarding disease control, as well as type and dose of medication. The reason for the absence of DR in some patients despite their high HbA1c is that the progression or severity of DR is not solely related with HbA1c. Rather, for DR to develop and to progress, other risk factors for retinopathy also need to be considered.

In the United Kingdom Prospective Diabetes Study (UKPDS) [49], development of DR was strongly associated with baseline glycaemia, glycemic exposure over 6 years, higher blood pressure and being a non smoker. In those who already had retinopathy, progression was associated with male sex, hyper glycaemia (as evidenced by a higher HbA1c), older age, and being a non smoker.

According to clinical practice guidelines on management of Type 2 DM 2015 [50], the modifiable risk factor of development and severity of DR are tight control of blood glucose, serum lipids and blood pressure, a proper diet, exercise and smoking cessation. However most of the factors above were not assessed in our study. These factors may be some of the reasons for development of DR in one group and not in the other. Our results highlight the need for good glycemic control if DR is to be minimised.

Our study found that there was no significant correlation between HbA1c and anterior ocular segment biometry, regardless of DR status. Our finding was comparable with study done by El-Agamy A and Alsubaie S showed that there was no correlation between CCT and HbA1c [8]. On the contrary, Su et al [16] previously found an association between HbA1c and CCT, and Yazgan S et al [6] recently showed there was a strong correlation between CCT and HbA1c. Amerasinghe et al [17] reported no association of angle parameters with HbA1c. However, studies done by Su et al [16] and Amerasinghe et al [17] were comparing diabetics with non diabetics, while our study is comparing these parameters within a group of diabetic patients. With regards to the ACW, there was no study done in correlating it with HbA1c.

In view of the significant differences in mean CCT which we noted between the DM and non DM groups, as well as significant differences in mean ACW between DM with NPDR and DM with no DR, we postulate that these differences may arise due to factors other than those analysed in this study, which may include the severity of the retinopathy, the duration of diabetes and the long-term control of diabetes over the previous years, which may not be adequately depicted by a single HbA1c reading. Anterior segment biometry parameters change over time; one HbA1c parameter only shows us one point in time, but does not give us any indication of the cumulative oxidative stress levels in this diabetic patient. We were not able to assess markers of oxidative stress in this case due to limited resources. Hence, large additional studies are needed in order to provide further explanation and support our findings.

Assessment and recognition of the anterior ocular segment biometry of diabetic patient is very important for treatments strategies in cases where ocular surgery is needed especially in laser refractive surgery. This is important because laser refractive surgery places the diabetic cornea at a higher risk, predominantly due to concerns that the comorbid ocular conditions associated with the disease might lead to poor refractive outcomes and significant postoperative complications [51,52]. However, some authors have observed that patients with well-controlled diabetes present good refractive outcomes [53,54]. The risk of complications after laser refractive surgery is related to poor glycemic control and laser refractive surgery may be safe in patients with no systemic or ocular complications [55].

AS-OCT is a non-contact imaging tool that provides the detailed structure of the anterior part of the eyes and quantitative analysis of the ocular tissues [56]. The clinical applications of AS-OCT depend on the reproducibility of its measurements. A good reproducibility means that the measurements obtained are not dependent on the operator [57]. The CCT measurement by ultrasound pachymetry gives higher values compared to AS-OCT measurements. Hence, they cannot be interchangeably used in clinical practice [58]. Studies showed that though both devices have good repeatability, AS-OCT has slightly better repeatability than ultrasound pachymetry [59,60]. Corneal and anterior segment OCT and AS-OCT provided comparable and well- correlated anterior ocular biometric measurements with sufficient repeatability and reproducibility [61].

Based on our research, we can conclude that anterior segment biometry varies between diabetic and non-diabetic patients, with the mean CCT being significantly thicker in diabetic patients. However, the presence of NPDR has no statistically significant effect on this parameter. As the mean ACW was significantly narrower in DM with NPDR as compared to DM with no DR, further research may reveal the pathogenesis of these differences as no published study done in comparing this parameter within diabetic groups.

Although, as expected, the higher median level of HbA1c was observed in the group of DM with NPDR as compared to DM with no DR, with a significant difference of HbA1c level between these groups, HbA1c was not found to be correlated with any parameters of anterior ocular segment biometry, regardless of retinopathy status. Further surveys on large groups of diabetic patients who have had diabetes for a longer period of time as well as assessment of factors other than those analysed in this study seem to be necessary to provide further explanation regarding our findings.

## Conclusion

Diabetic patients appear to have significantly thicker CCT regardless of retinopathy status whereas ACW was significantly narrower in diabetic with NPDR group compared to diabetic with no DR. However, there was no significant correlations between all anterior ocular segment biometry and HbA1c in diabetic patients regardless of diabetic retinopathy status.

## Abbreviations

ACA: anterior chamber angle
ACG: Angle closure glaucoma
ACW: anterior chamber width
AOD: angle opening distance
AS-OCT: Anterior segment optical coherence tomography
CCT: central corneal thickness
CI: confidence interval
DM: Diabetes mellitus
DR: Diabetic retinopathy
EDTA: Ethylenediaminetetraacetic acid
ETDRS: Early Treatment Diabetic Retinopathy Study
HbA1c: Glycosylated haemoglobin
NHMS: National Health Morbidity Survey
NPDR: Non proliferative diabetic retinopathy
OCT: Optical coherence tomography
PACG: Primary angle closure glaucoma
PDR: Proliferative diabetic retinopathy
PS: Power and sample size
SD: Standard deviation
SPSS: Statistical Package for Social Sciences
UKPDS: United Kingdom Prospective Diabetes Study
